# A Predictive Model of Postnatal Surgical Intervention in Children With Prenatally Detected Congenital Anomalies of the Kidney and Urinary Tract

**DOI:** 10.3389/fped.2019.00120

**Published:** 2019-04-02

**Authors:** Mariana A. Vasconcelos, Eduardo A. Oliveira, Ana Cristina Simões e Silva, Cristiane S. Dias, Robert H. Mak, Carolina C. Fonseca, Ana Paula M. Campos, Ewout W. Steyerberg, Yvonne Vergouwe

**Affiliations:** ^1^Pediatric Nephrology Unit, Department of Pediatrics, Federal University of Minas Gerais (UFMG), Belo Horizonte, Brazil; ^2^Division of Pediatric Nephrology, Rady Children's Hospital San Diego, University of California, San Diego, San Diego, CA, United States; ^3^National Institute of Science and Technology (INCT) of Molecular Medicine, Belo Horizonte, Brazil; ^4^Department of Pediatrics, Federal University of Minas Gerais (UFMG), Belo Horizonte, Brazil; ^5^Department of Public Health, Erasmus University Medical Center, Rotterdam, Netherlands

**Keywords:** congenital anomalies of kidney and urinary tract, prenatal diagnosis, antenatal hydronephrosis, surgical intervention, predictive model

## Abstract

The aim of this study was to identify predictive factors and develop a model to assess individualized risk of postnatal surgical intervention in patients with antenatal hydronephrosis. This is a retrospective cohort study of 694 infants with prenatally detected congenital anomalies of kidney and urinary tract with a median follow-up time of 37 months. The main event of interest was postnatal surgical intervention. A predictive model was developed using Cox model with internal validation by bootstrap technique. Of 694 patients, 164 (24%) infants underwent surgical intervention in a median age of 7.8 months. Predictors of the surgical intervention in the model were: baseline glomerular filtration rate, associated hydronephrosis, presence of renal damage and the severity of renal pelvic dilatation. The optimism corrected c statistic for the model was 0.84 (95%CI, 0.82–0.87). The predictive model may contribute to identify infants at high risk for surgical intervention. Further studies are necessary to validate the model in patients from other settings.

## Introduction

Antenatal hydronephrosis (ANH) is one of the most common birth defects and is a surrogate marker of potential congenital renal anomalies (1, 2). Congenital anomalies of the kidney and urinary tract (CAKUT) comprise a wide phenotypic spectrum and are important causes of kidney morbidity and the most frequent cause of chronic kidney disease (CKD) and end-stage renal disease in infants and young children (3–5).

The prenatal detection of CAKUT has permitted a refinement of the management of these conditions (6–11). Nevertheless, in spite of the continuous advances in the understanding of the genetic basis and outcomes of CAKUT, there are still many controversies regarding postnatal evaluation and management of infants with ANH. Consequently, taken into account the heterogeneity of CAKUT, there is an understandable little consensus about the best approach for these patients (12). In this setting, a relevant issue concerning the management of CAKUT is to establish a consistent approach to discern which patients would benefit from surgical intervention and which may be best assisted by continued surveillance (2, 13). Retrospective cohort studies have suggested some predictive factors for surgery or for spontaneous resolution of renal pelvic dilatation (14, 15), including the magnitude of renal pelvic diameter, the Society of Fetal Urology (SFU) grading system, renal parenchyma-to-hydronephrosis area ratio, renal cortical thickness, and renal function on renal scintigraphy (16–18).

We have previously described the clinical course of children with prenatally detected CAKUT and we identified variables that are possible predictors of progression to CKD (5, 19). The aim of this retrospective cohort study was to identify potential predictive factors and to develop a model to assess individualized risk of postnatal surgical intervention in patients with antenatal detected CAKUT.

## Patients and Methods

### Patients

All infants with diagnosis of CAKUT (*n* = 819) admitted at the Pediatric Nephrology Unit (Hospital das Clínicas, Federal University of Minas Gerais (UFMG), Brazil) from 1987 to 2013 were followed-up. Patients with aneuploidy, multiple malformations, neurogenic bladder or loss of follow-up soon after the birth were excluded (*n* = 125). In total, 694 infants were included in the analysis.

### Clinical Protocol

During the 25 years of this study, the clinical protocol for the management of infants with perinatal diagnosis of CAKUT has inevitably evolved. In the first decade of the study, infants were investigated according to a comprehensive systematic protocol. Briefly, all patients with anterior posterior renal pelvic diameter (APRPD) ≥5 mm were placed on prophylactic antibiotics at birth and submitted to an extensive imaging protocol, including renal ultrasonography (RUS), voiding cystourethrogram (VCUG), and renal scintigraphy. After 2000, we developed a more tailored clinical protocol, based mainly on the severity of the renal pelvic dilatation (20). All infants underwent VCUG within 3 months of life until 2009. Since then, VCUG has been indicated for a selected subgroup of patients with fetal or postnatal APRPD > 10 mm and or ureter dilatation (21). Renal scintigraphy (^Tc−99m^DMSA and ^Tc−99m^DTPA) was performed after the first month of life for patients with APRPD ≥10 mm (22). Antibiotic prophylaxis was started on the first postnatal day and maintained in accordance with the postnatal diagnosis (23).

### Follow-Up Protocol

After initial clinical and imaging evaluation, RUS scans, clinical visits, and laboratory reviews (including urine culture and serum creatinine) were scheduled at 6-month intervals. In short, the clinical approach consisted of full physical examination, including evaluation of anthropometric measurements and blood pressure performed at 6-month intervals. Urine cultures were obtained at each 6-month follow-up visit, and it was recommended that urine samples be collected during any unexplained febrile episode or in the presence of urinary symptoms.

### Outcome

The main event of interest was defined as the time from birth until the first surgical intervention.

### Candidate Predictors

The following variables at baseline were considered in the analysis: gender, age, serum creatinine, the estimative of glomerular filtration rate (eGFR) based on conventional or modified Schwartz formulas (24, 25), oligohydramnios, presence of other ultrasonographic urinary tract alterations besides antenatal hydronephrosis (associated hydronephrosis), renal pelvic dilatation (RPD) laterality (unilateral vs. bilateral), presence of renal damage (RD) on ^Tc−99m^DMSA scan and APRPD. Combined data obtained by VCUG, renal scan and sequential RUS were considered for the diagnosis of urinary tract anomalies. The absence of any recognized uropathy was classified as idiopathic hydronephrosis. Isolated hydronephrosis was defined as the presence of APRPD ≥5 mm without any other alterations of the urinary tract. Associated hydronephrosis was defined as the presence of APRPD >5 mm combined with other alterations, including megaureter and megacystis. When bilateral renal pelvic dilation was present, the largest APRPD was considered for analysis. Presence of RD on ^Tc−99m^DMSA scan was classified as none, unilateral and bilateral, when there was renal scarring and/or parenchymal atrophy in none, one or both kidneys, respectively. The diagnosis of oligohydramnios was based on amniotic fluid index (26). Since creatinine measurements were made using the Jaffe method until November 2011 in our institution, glomerular filtration rate (eGFR) was estimated by the conventional Schwartz formula (24) for data obtained until this period. After November 2011, creatinine was measured using the isotope dilution mass spectrometry (IDMS) traceable method and, for this reason, we adopted the modified Schwartz formula (25).

### Statistical Analysis and Development of the Risk Prediction Model

The values are expressed as medians and interquartile ranges (IQs) or frequencies when appropriate. Survival analyses were performed by the Kaplan–Meier method (KM) and by the Cox proportional hazards model to evaluate time until the occurrence of the event. Differences between dichotomous variables were assessed by the two-sided log-rank test. The Cox proportional hazards model was applied to identify variables that were independently associated with the occurrence of the event. Variables selected for multivariable analyses were used to build a final model after discarded any violation of proportionality assumptions. Cox proportional hazards regression analysis was used to assess the association between the candidate predictors and the occurrence of surgery. Hazard ratios for continuous variables were given for the 75 percentile vs. 25 percentile of the variable. Using a backward elimination strategy with *p* < 0.1, the strongest prognostic factors were included in the final model (27).

Missing values for candidate predictors were filled in with multiple imputation (MI) procedure. Each missing value was imputed five times. Imputed values were drawn from the predictive distribution in an imputation model that included all candidate predictors and the outcome (time to surgery). MI resulted in five complete datasets, which were analyzed with standard complete data methods. The results were combined to produce overall estimates and standard errors that reflect missing data uncertainty (28).

When prediction models are developed in relatively small samples, they may be overfitted and may show optimistic performance. To adjust for overfitting and optimistic performance of the model, we used bootstrap resampling for internal validation. One hundred bootstrap samples were drawn with replacement; a prognostic model was developed in each sample; and the performance was evaluated in the bootstrap sample and in the original sample (29). The model was presented as a nomogram where each predictor could be judged for its relative importance by the number of points attributed over the range of the predictor (30). Statistical analyses were performed with R software version 2.13.1 (R Foundation for Statistical Computing, Vienna, Austria) and SPSS version 18.0 (SPSS, Inc., Chicago).

## Results

The main baseline clinical characteristics of 694 patients included in the analysis are summarized in Table 1. The median follow-up time of the cohort was 37 months (Interquartile range (IQ), 14–85 months). A total of 164 (24%) patients underwent surgery at a median age of 7.8 months (IQ, 2.4–16.1 months). The majority of patients were males, considering the entire CAKUT group (65%) as well the subgroup of children who underwent surgical intervention (76%).

**Table 1:** Baseline clinical characteristics of 624 infants included in the analysis.

	***N* (%)**
**GENDER**	
Male	453 (65.3)
Female	241 (34.7)
**LATERALITY—FETAL US**	
Unilateral	411 (59.3)
Bilateral	283 (40.7)
**PERIOD**	
1989–1999	174 (25.0)
2000–2013	520 (75.0)
**POSTNATAL DIAGNOSIS**	
Idiopathic hydronephrosis	277 (39.9)
Ureteropelvic obstruction junction	122 (17.6)
Multicystic dysplastic kidney	97(14)
Vesicoureteral reflux	57 (8.2)
Primary megaureter	40 (5.8)
Posterior urethral valves	26 (3.7)
Ureterocele	20 (2.9)
Hypodysplastic kidney	14 (2.0)
Prune-belly syndrome	6 (0.9)
Others	35 (5.0)

Table 2 shows the baseline findings and the association between patient characteristics and postnatal surgical intervention. In univariate analysis, the following variables were associated with the outcome: age, serum creatinine, eGFR, oligohydramnios, associated hydronephrosis, laterality, presence of RD, and APRPD.

**Table 2:** Patients' characteristics and association with surgical intervention.

**Characteristics**	**Unit or category**	**Surgical intervention**		**Hazard ratio (95%CI)**
		**Yes** ***N* = 164**	**No** ***N* = 530**	
Gender	Male/female	124 (76%)	329 (62%)	1.2 (0.82–1.6)
Age at admission	Months	1.1 (0.19–2.9)	2.2 (0.82–4.9)	1.1 (1.0–1.2)^#^
Plasma creatinine	mg/dl	0.50 (0.39–0.73)	0.36 (0.30–0.50)	1.2 (1.1–1.2)
eGRF	ml/min/1.73 m^2^	52(31–75)	71 (51–92)	2.2 (1.7–2.9)^#^
Oligohydramnios^*^	Yes/no	14 (8.5%)	12 (2.3%)	3.7 (2.1–6.4)
Assoc. hydro^**^	Yes	92 (61%)	160 (31%)	3.0 (2.2–4.2)
Laterality	Uni/bilateral	63 (45%)	220 (43%)	1.2 (0.83–1.6)
Renal damage^***^	None	55 (38%)	200 (69%)	Reference
	Unilateral	75 (52%)	84 (28%)	2.6 (1.9–3.7)
	Bilateral	13 (10%)	9 (3%)	4.3 (2.3–7.8)
APRPD^****^	mm	20(13-29)	8(6–12)	1.5 (1.4–1.7)

*eGRF, estimated glomerular renal function; APRPD, anteroposterior renal pelvis dilatation; RPD, renal pelvic dilatation; 95%CI, 95% confidence interval. Values are given as number (percentage of observed total) or median (25–75 percentile), unless stated otherwise; Hazard ratios for continuous variables are given for the 75 percentile vs. the 25 percentile*.

* *missing, 426*;

** *missing, 25*;

*** *missing, 258*;

**** *missing, 85*.

# *risk is higher for lower predictor values*.

**Figure 1:**
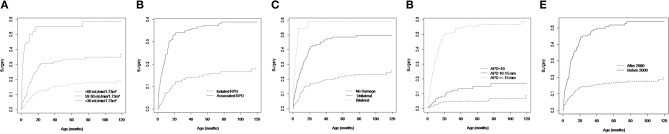
Kaplan Meier estimates the risk of surgical intervention stratified according to **(A)** renal function at baseline; **(B)** associated hydronephrosis; **(C)** renal damage; **(D)** renal pelvic dilatation; and **(E)** period of admission.

After adjustment by the Cox model, four predictors had strong effects and remained in the final model: eGFR, associated hydronephrosis, renal damage, and severity of renal pelvic dilatation (Table 3). Figures 1A–E shows the Kaplan-Meier survival curves for the predictive variables of postnatal surgical intervention. The uniform shrinkage factor estimated with bootstrapping was 1.0. The *c* statistic was 0.81 (95% CI 0.77–0.83), after correction for optimism.

**Table 3:** Multivariable association of the selected predictive factors for surgical intervention.

**Predictive factor**	**Unit or category**	**Hazard ratio [95% CI]**
eGFR	47 vs. 90 ml/min/1.73 m^2^	1.7 (1.3–2.2)
Associated Hydro.	Yes/no	1.9 (1.3–2.6)
Renal damage	Unilateral	2.3 (1.6–3.2)
	Bilateral	4.0 (2.1–7.4)
APRPD	16 vs.7 mm	1.4 (1.3–1.5)

*Hazard ratios for continuous variables are given for the 75 percentile vs. the 25 percentile. eGRF, estimated glomerular renal function; APRPD, anterior posterior renal pelvis diameter; RPD, renal pelvic dilatation*.

**Figure 2:**
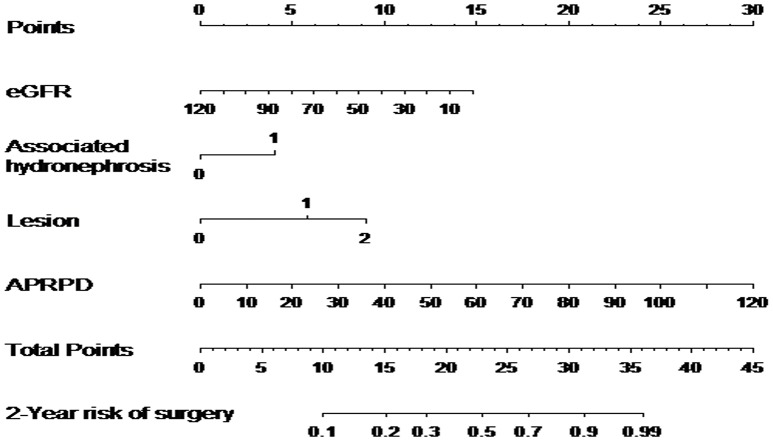
Nomogram for predicting 2 years risk of surgery based on 5 predictors; estimated glomerular renal function in mL/min/1.73 m^2^ (eGFR), associated hydronephrosis, renal damage, APRPD (anteroposterior renal pelvis diameter) in mm, period of admission (before 2000); 2-year risk of surgery.

The predictive model is presented as nomogram to provide the risk that a patient undergoes surgical intervention within 2 years (Figure 2). To use the nomogram, a line from each predictor value needs to be drawn upwards to the point axis. Then, the points corresponding to the predictor values need to be added and the result to be located to the Total Points axis. A line from the total points value to the axis for 2 years risk of surgery needs to be drawn to find the risk of surgery within 2 years of follow-up.

The formula with shrunk coefficients used to calculate the individual absolute risks of postnatal surgical intervention was calculated as follow:

$$\text{risk(t)}=\left[1-S_{0}{\left(t\right)}^{\text{exp}(lp)}\right]\;\times \;100\%,\text{ where}$$

*lp* = − 0.012 × eGFR + 0.62 × associated hydronephrosis +0.83 × RD uni + 1.37 × RD bi + 0.037 × APRPD; S_0_(12) = 0.962; S_0_(36) = 0.920; S_0_(60) = 0.910.

For example, a child with eGRF of 40 ml/min/1.73 m^2^ (10 points), associated hydronephrosis (5 points), bilateral RD (9 points), APRPD of 40 mm (10 points). The total of 34 points corresponds to a more than 90% of risk of surgical intervention before 2 years age.

**Figure 3:**
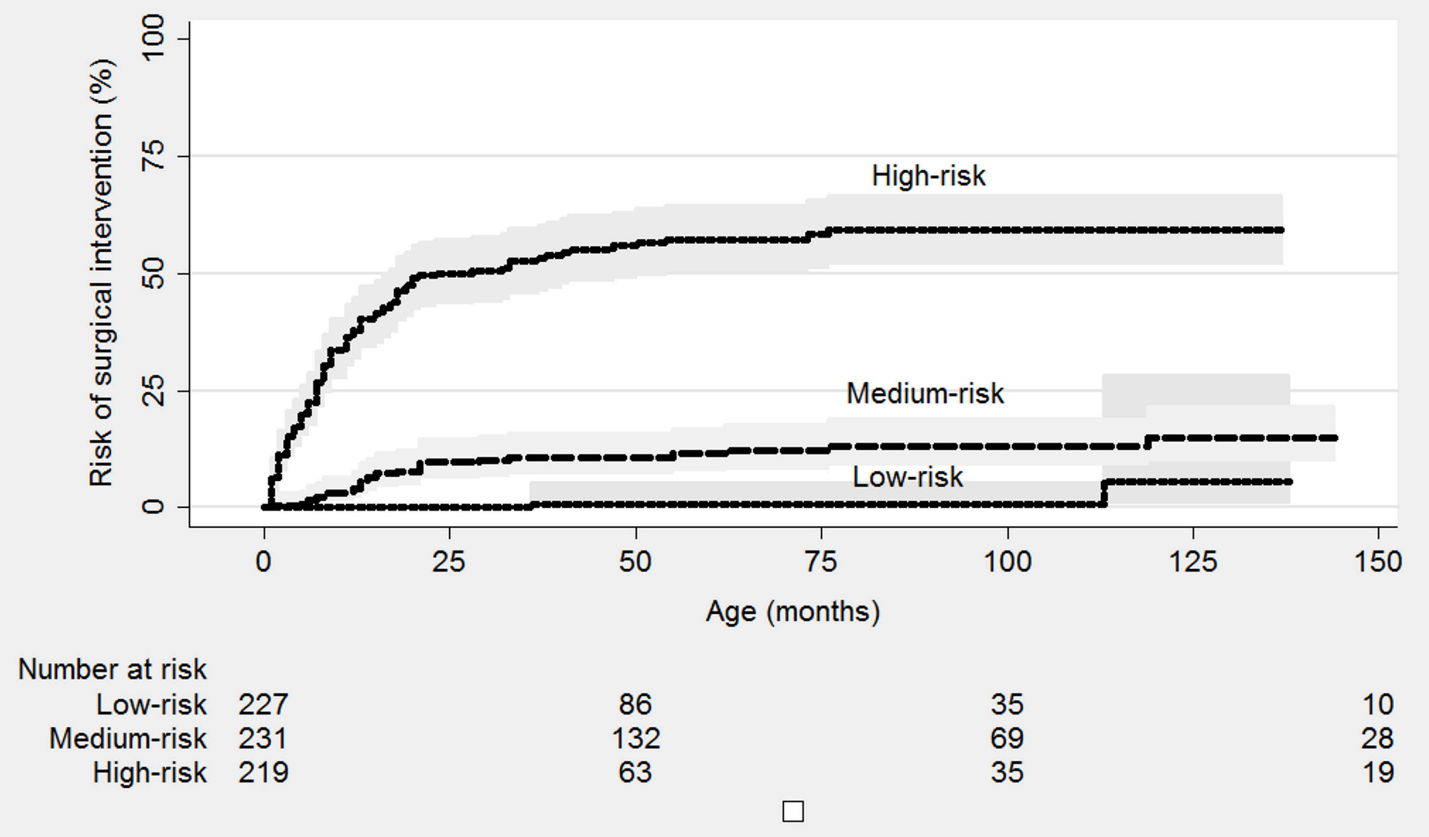
Kaplan Meier plot for risk of surgery according to risk category. Low-risk category, <20 points; Medium-risk category, 20–28 points; High-risk category, 29+ points.

**Figure 4:**
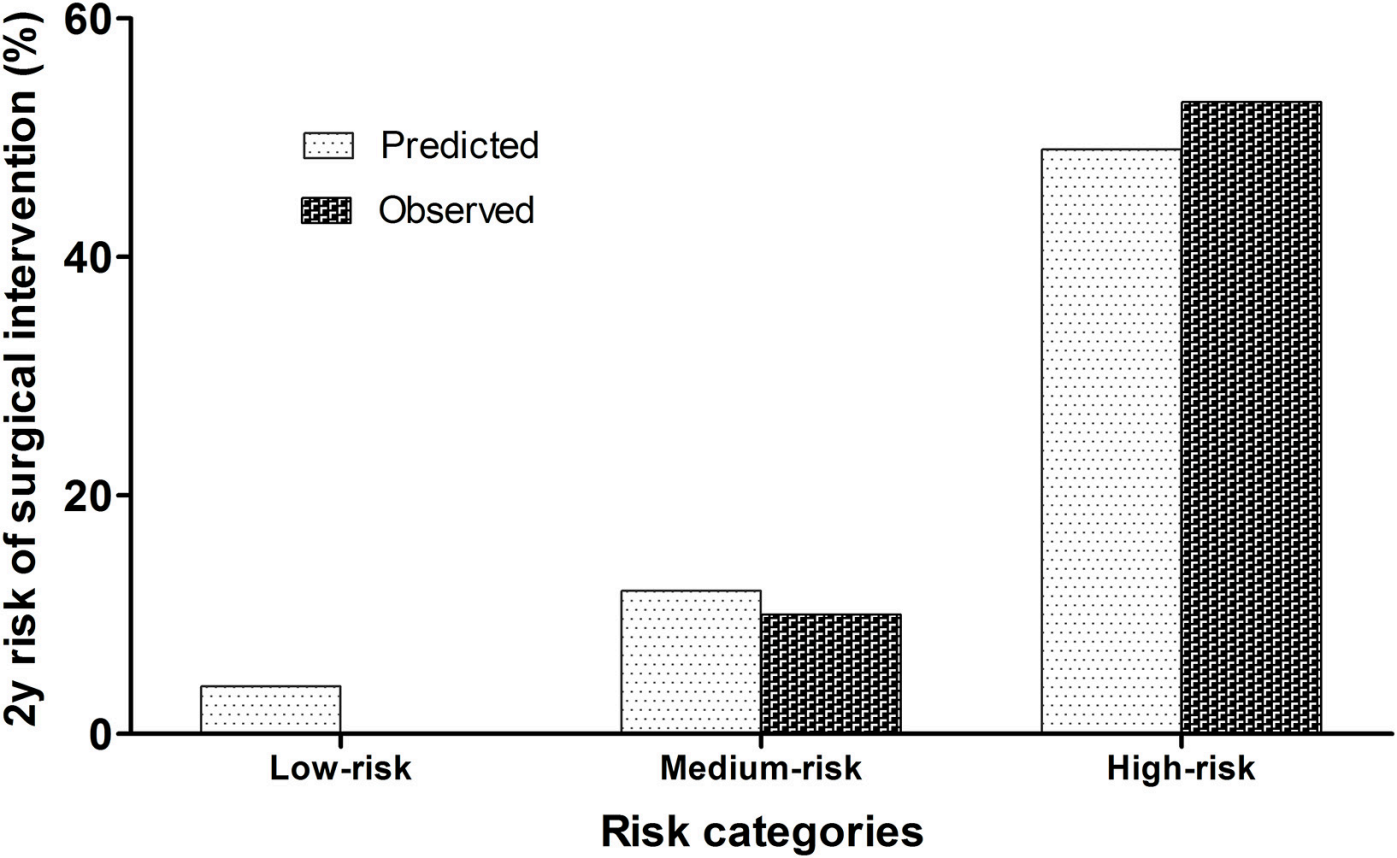
Agreement between predicted 2-year risk of surgery and observed risk.

Finally, the sample was divided into three risk scores: low-risk (<20 points), medium-risk (20–28 points) and high-risk (≥29 points). The risk of surgical intervention at 2 years of age was estimated as 10% for patients assigned to the medium-risk group and 53% for patients of the high-risk group (*P* < 0.001, Figure 3). Figure 4 shows the calibration plots for the model of risk prediction during 2 years of follow-up. The model showed good calibration for all risk categories although with a slight overestimated risk of surgical intervention for low and medium-risk groups.

## Discussion

In this cohort study, we evaluated predictive factors of surgical intervention in a large cohort of children with CAKUT enrolled at a tertiary center between 1987 and 2013. Overall, about a quarter of these patients underwent surgical intervention. Nevertheless, children born after 2000 had surgical intervention less frequently (15%). After adjustment by Cox multivariate model, four variables remained as predictive factors for surgical intervention: eGFR, associated hydronephrosis, presence of RD on baseline DMSA scan and the APRPD. Finally, we combined the factors in a clinical predictive model for surgical intervention with a good predictive performance.

CAKUT is a heterogeneous and complex group of diseases associated with UTI, CKD and hypertension in children (31–34). Antenatal ultrasonography reveals an increasing number of renal anomalies in otherwise uncomplicated pregnancies. Thus, antenatal screening creates a clinical group of patients, often asymptomatic, for whom postnatal management and follow-up are required (35, 36). The main goal of fetal screening for uropathies is to prevent complications. In this regard, the majority of CAKUT is non-surgically managed, but, in some cases, surgery will be necessary to avoid renal parenchyma damage and recurrent urinary tract infections. Currently, nonsurgical management of CAKUT should be considered whenever possible for infants with ANH. Nevertheless, there are still controversies regarding the best postnatal approach for many uropathies (34, 36–38). Unnecessary interventions must be avoided, but late procedures may enhance the risk of infection and renal parenchymal loss (34). However, the ability to define which children will resolve their condition or will benefit from a surgical procedure remains elusive. So far, a single reference test is not available to define which patient and at what time will need surgery. Early identification of patients at high risk for surgical procedures may be helpful for medical decisions and may decrease unnecessary interventions in low risk patients (37, 39). Moreover, prenatal diagnosis studies have shown that families are worried irrespective of suspicious of a mild hydronephrosis or of a severe malformation. The information about fetal malformation findings during the ultrasound examination often comes unexpectedly, and families may not necessarily receive at the time of diagnosis any conclusive statement on the prognosis (40). Thus, clinical predictive models can enhance the ability of the health team to manage this stressful situation.

As expected, in our series, patients admitted at the first period of the study (1983–1999) had a greater probability to undergo surgical intervention (RR = 4.2, 95%CI, 2.8–6.3). The prenatal detection of CAKUT has permitted a refinement of the management of these conditions. For instance, it has been clearly demonstrated by longitudinal studies that many children with partial ureteropelvic or ureterovesical junction obstruction will spontaneously resolve their hydronephrosis (31, 41). Consequently, our findings probably reflected the shift on the management of asymptomatic infants with antenatal hydronephrosis. Currently, there is a consensus that a nonsurgical management should be considered whenever possible for infants with antenatal hydronephrosis (13, 36, 37).

Previous studies have also identified predictive factors of surgical intervention in patients with CAKUT (15, 39) For instance, Nef et al. (39) reported impaired renal function, oligohydramnios and postnatal bilateral renal anomalies as potential predictors of surgical intervention. The latter two were also associated with surgical intervention in the current cohort, but not included in the final model.

Post-natally, the most common measurements of urinary tract dilation (UTD) are APRPD and the SFU grading system (8). An accurate description of the degree of UTD is important because its severity can guide further management. Severe fetal UTD is commonly associated with significant postnatal uropathy, often requiring surgery (42). Unfortunately, due to the retrospective design of our study, we were not able to use the SFU system as a possible predictive factor in the model. Nevertheless, the magnitude of renal pelvic dilatation was an independently predictive factor for surgical intervention in the final model. A number of studies have shown the good performance of measurement of APRPD as a predictor of the severity of the renal anomaly, especially in the context of the isolated antenatal hydronephrosis (15, 16, 22). Recently, Arora et al. (14) reported a prospective single-center study including 122 renal units with ANH. A multivariate analysis revealed APRPD and preoperative differential renal function on renal scintigraphy as the only independent predictors for surgical intervention. As expected, in our series, renal damage on renal scintigraphy was also a predictor of surgical intervention. Renal scintigraphy has been the most commonly used modality to determine the presence of upper urinary tract obstruction in infants with ANH. However, the diagnostic accuracy of diuretic renal scintigraphy has been reported to be highly variable. In a notable review, Ismaili and Piepsz (43) outlined the advances, pitfalls, and difficulties in the interpretation of renography for the evaluation of upper tract obstruction in infants. Interestingly, in a well-designed study, the same group concluded that diuretic renal scintigraphy should only be performed in patients with APRPD more than 30 mm, major calyceal dilatation, and/or parenchymal thinning (44).

Some clinical and methodological considerations should be taken into account in evaluating our findings. From the methodological point of view, we did not validate this risk prediction instrument in an independent cohort. External validation is important, because accurate predictions in our cohort do not necessarily guarantee good accuracy in other patients (45). Missing data are a common problem in all retrospective cohort studies. We have addressed this problem by using multiple imputations that have the advantage of not ignoring observations with incomplete information and, at the same time, reflecting the uncertainty of the imputation process. Regarding methodological clinical aspects, a weakness is that the medical team decided the surgical intervention based mainly on the variables in the model. This fact would imply that we modeled the decision making of the surgery rather than the “real” need of surgery. However, it must be pointed out that the children who did not undergo surgery had a long follow-up time in our series and the medical treatment was shown as a safe approach for these patients. Another fragility is that false positive cases, i.e., children who underwent surgery but was not benefited by it, could not be identified due to the observational design of our study. In addition, our sample contains mixed phenotypes of CAKUT, including both isolated hydronephrosis and more complex entities, which may interfere with accurate predictions. On the other hand, some features of our study may increase the strength of our findings, including the large dataset collected over many years, the length of the follow-up time, and the management by the same medical team using a standardized protocol.

In summary, we have developed a clinical predictive model of surgical intervention in children with CAKUT. The magnitude of renal pelvic dilatation, renal function at baseline, presence of associated hydronephrosis and of renal damage are independent predictors of surgical intervention during follow-up. This clinical predictive model, if confirmed in future studies and after external validation, may help the medical team to identify infants with antenatal hydronephrosis at high-risk for surgical intervention during the first years of life.

## Data Availability

The datasets generated for this study are available on request to the corresponding author.

## Ethics Statement

The study was approved by the Ethics Committee of our institution. Parents or legal guardians responsible for the children gave written informed consent to participate.

This study was carried out in accordance with the recommendations of Ethics Committee of the UFMG with written informed consent from all subjects. All subjects gave written informed consent in accordance with the Declaration of Helsinki. The protocol was approved by the Ethics Committee of the UFMG.

## Author Contributions

EO and RM: research idea and study design. MV, AS, CF, AC, and CD: data acquisition. MV, EO, AS, RM, ES, and YV: data analysis/interpretation. MV, ES, and YV: statistical analysis. EO and AS: supervision or mentorship. Each author contributed important intellectual content during manuscript drafting or revision and accepts accountability for the overall work by ensuring that questions pertaining to the accuracy or integrity of any portion of the work are appropriately investigated and resolved. EO takes responsibility that this study has been reported honestly, accurately, and transparently; that no important aspects of the study have been omitted; and that any discrepancies from the study as planned (and, if relevant, registered) have been explained.

### Conflict of Interest Statement

The authors declare that the research was conducted in the absence of any commercial or financial relationships that could be construed as a potential conflict of interest.
